# Reducing Wildlife Damage with Cost-Effective Management Programmes

**DOI:** 10.1371/journal.pone.0146765

**Published:** 2016-01-15

**Authors:** Cheryl R. Krull, Margaret C. Stanley, Bruce R. Burns, David Choquenot, Thomas R. Etherington

**Affiliations:** 1 Institute for Applied Ecology New Zealand, AUT University, Auckland, New Zealand; 2 Centre for Biodiversity and Biosecurity, University of Auckland, Auckland, New Zealand; 3 Institute for Applied Ecology, University of Canberra, Canberra, ACT, Australia; 4 Royal Botanic Gardens Kew, Wakehurst Place, Ardingly, United Kingdom; 5 School of Environment, University of Auckland, Auckland, New Zealand; University of Tasmania, AUSTRALIA

## Abstract

Limiting the impact of wildlife damage in a cost effective manner requires an understanding of how control inputs change the occurrence of damage through their effect on animal density. Despite this, there are few studies linking wildlife management (control), with changes in animal abundance and prevailing levels of wildlife damage. We use the impact and management of wild pigs as a case study to demonstrate this linkage. Ground disturbance by wild pigs has become a conservation issue of global concern because of its potential effects on successional changes in vegetation structure and composition, habitat for other species, and functional soil properties. In this study, we used a 3-year pig control programme (ground hunting) undertaken in a temperate rainforest area of northern New Zealand to evaluate effects on pig abundance, and patterns and rates of ground disturbance and ground disturbance recovery and the cost effectiveness of differing control strategies. Control reduced pig densities by over a third of the estimated carrying capacity, but more than halved average prevailing ground disturbance. Rates of new ground disturbance accelerated with increasing pig density, while rates of ground disturbance recovery were not related to prevailing pig density. Stochastic simulation models based on the measured relationships between control, pig density and rate of ground disturbance and recovery indicated that control could reduce ground disturbance substantially. However, the rate at which prevailing ground disturbance was reduced diminished rapidly as more intense, and hence expensive, pig control regimes were simulated. The model produced in this study provides a framework that links conservation of indigenous ecological communities to control inputs through the reduction of wildlife damage and suggests that managers should consider carefully the marginal cost of higher investment in wildlife damage control, relative to its marginal conservation return.

## Introduction

Wildlife, both native and invasive, may cause damage to natural and agricultural ecosystems, fisheries, urban areas and human/animal health [1]. The global costs of wildlife damage are difficult to quantify but there is no doubt that economic losses are significant [2]. The challenge for managers becomes reducing the extent of wildlife damage and the cost, through the control of overabundant wildlife populations. Controlling wildlife in order to limit the impact of their damage requires an understanding of how control inputs change the occurrence of damage, through their effect on prevailing animal density. The underlying theoretical relationship between animal density and the extent of damage can be thought of as a “damage function” [3].

In this study we use wild pig disturbance and management to demonstrate the importance of linking the damage function to management inputs. Wild pigs (*Sus scrofa*) are a major threat to biodiversity conservation throughout their native and introduced range [4–8]. While predation on a range of threatened species can lead to critical local problems [9–12], widespread ground disturbance caused by pigs is of more global biodiversity concern [6]. Ground disturbance significantly modifies habitats by affecting establishment and growth of plants and alters ecosystem structure [13–18], facilitating spread of invasive weeds [19–23], modifying soil traits [24–25, 18], and can lead to competition with native animals for food resources [24, 26]. Research has also highlighted the role pigs potentially play in the spread of soil-borne plant pathogens [27–28].

Despite concerns about the immediate and cumulative impacts of ground disturbance, research linking changes in pig density to changes in ground disturbance (the damage function) is limited [6, 8] and the general assumption is that a reduction in pig density will result in an equal reduction in ground disturbance. We measured changes in ground disturbance over a three-year wild pig control programme undertaken in a temperate rainforest area in New Zealand. Combining these data with information collected during the control programme, we explored the effect control had on pig abundance, rate of ground disturbance and rate of ground disturbance recovery. The relationships produced were used to estimate parameters for a simulation model linking control to rates of ground disturbance and recovery. The model was used to investigate the cost and effectiveness of alternative strategies to limit the extent of ground disturbance.

## Materials and Methods

### Study site

**Fig 1:**
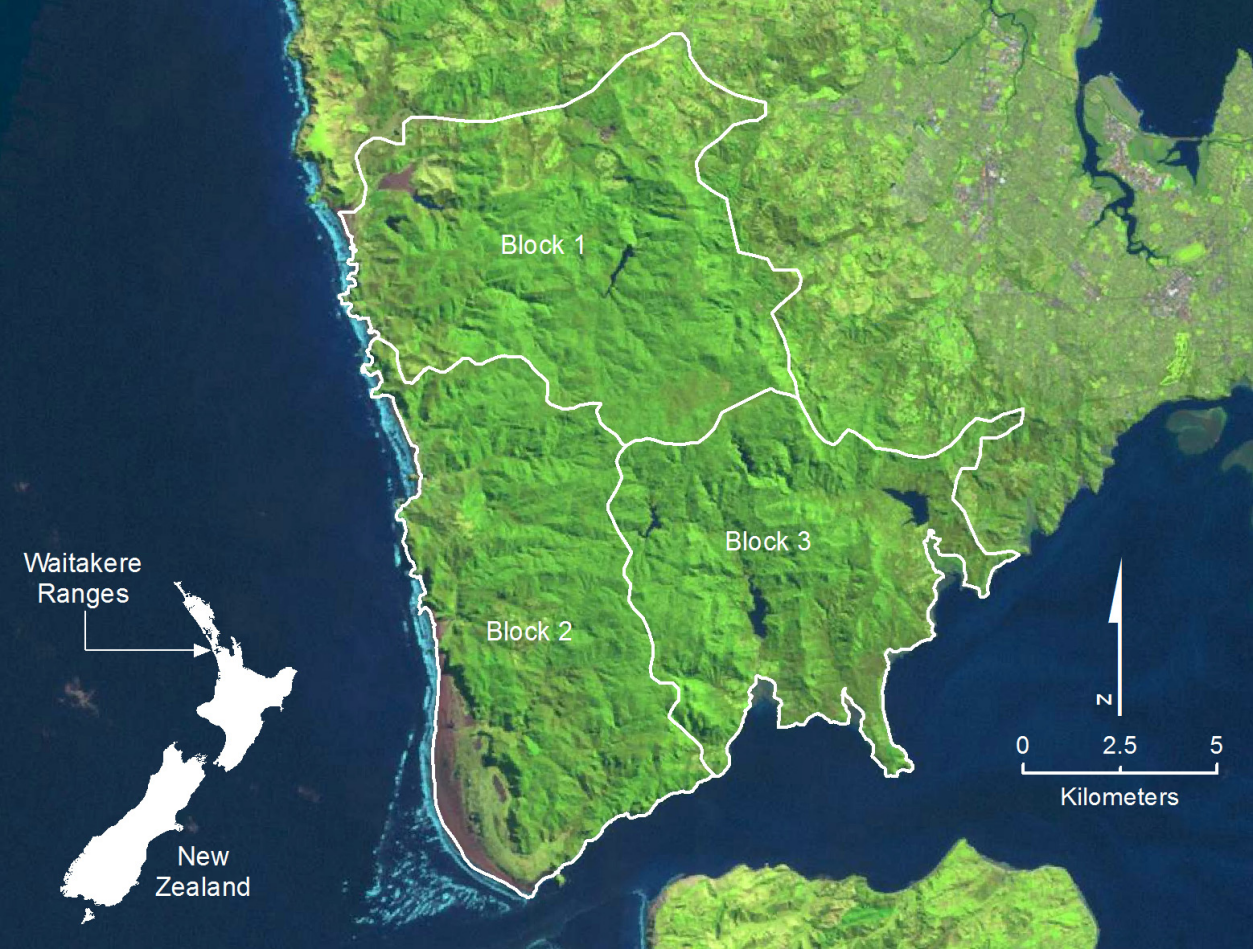
Location of the Waitakere Ranges study site on the North Island of New Zealand. The study site was divided into three hunting blocks for the purposes of organising the pig control programme. The Landsat image is sourced from the U.S. Geological Survey’s EarthExplorer http://earthexplorer.usgs.gov/ and is freely available public domain data. The outline of New Zealand is sourced from the LINZ Data Service https://data.linz.govt.nz/ and is licensed by LINZ for re-use under the Creative Commons Attribution 3.0 New Zealand licence.

The Waitakere Ranges is a conservation park managed by Auckland Council, New Zealand (Fig 1), with elevation 0−474 m above sea level, mean annual rainfall of 1,240 mm, and average temperatures between 7−20° centigrade. Vegetation is dominated by podocarps and various broadleaved tree species [29]. The area is bounded by peri-urban areas and ocean, suggesting movement of pigs in and out is limited. Prior to this study, pig abundance in the area was considered high because recreational hunting is illegal. No other large mammals are present.

### Hunting programme

Animal ethics was not required under New Zealand law. The Auckland Council (the local authority) conducted all hunting for pest control purposes on local authority land. Full details of the hunting methods used by The Auckland Council are now described. The study area was divided into three hunting blocks of approximately equal size (Block 1 = 62.58 km^2^, Block 2 = 55.04 km^2^, Block 3 = 55.91 km^2^) (Fig 1), with teams of 2−5 professional hunters assigned to each. Hunting operations (n = 9; August 2008−2011) occurred at approximately 6-month intervals over the first 18 months of the study (3 operations), then at 3-month intervals (6 operations). Hunting was carried out using teams of trained dogs. Once bailed, pigs were dispatched with a knife or rifle. Hunting hours, and time and location of kills were recorded using a GPS. Hunting operations averaged 26 days (range: 17.2−38.4 days, standard error = 2.51). As both the area and duration of hunting varied between blocks, we expressed hunting success per km^2^ and hunting effort per year to create standardised measures for all blocks.

### Modelling pig density over the hunting programme

Pre-hunting pig density for the Waitakere Ranges was estimated using a standard catch-effort methodology [30–31]. This method estimates an initial population size by assuming a linear decline between the accumulated catch and the catch-per-unit-effort. To assess this assumption, linear, exponential, power, log, and second-order polynomial models were used to assess the observed trend. The models were evaluated using the coefficient of determination (*R*^2^) and Akaike weights (*w*) based on the second-order bias corrected Akaike information criterion (AICc) calculated from model residuals [32]. The mean and standard deviation of the initial population estimate were converted to geometric mean and standard deviation [33] in order to define a log-normal probability distribution describing the likely pre-hunting pig density.

Variation in pig density over the course of the hunting programme was then estimated by a simple discrete logistic population growth model with a monthly time interval that included the known density of pig removals (*C*) within a given month:

$$N_{t}=N_{t-1}+r_{m}N_{t-1}\left(\frac{1-N_{t-1}}{K}\right)-C_{t}$$ (1)

The maximum intrinsic rate of increase for pig populations (*r*_*m*_) was set at 0.7 based on estimates obtained by Choquenot [34] (*r*_*m*_ = 0.69), and by Hone [15] (*r*_*m*_ = 0.74), with similar values of *r*_*m*_ measured by Giles [35] (*r*_*m*_ = 0.6−0.7) and Hone [36] (*r*_*m*_ = 0.57). The population model assumed that the pre-hunting pig density estimate was a reasonable approximation of density at carrying capacity (*K*), and that while the initial population was at carrying capacity, *K* would alter annually to represent potential changes in environmental conditions.

To acknowledge the uncertainty in the estimates of *K*, we ran 1000 iterations of the model, with the initial and annual changes of *K* determined as a random selection from the log-normal distribution fitted to the initial population estimate. Pig densities over the duration of the hunting programme were then estimated as the mean of the 1000 model runs.

### Ground disturbance

Ground disturbance monitoring was conducted with permission from The Auckland Council. Transects were established to monitor ground disturbance over the duration of the study. The study area was divided into a 23 block (2.8 km^2^ each) grid, with a 200 m straight-line transect (randomly orientated) located at the centre of each block. Transects were monitored four times (November 2008, September 2009, December 2010 and August 2011) and on each occasion, the extent (m) of disturbance that occurred along the transect (within 1 m either side of a transect tape) was mapped and each occurrence of disturbance assigned an age (new, aged or old) based on the dampness of the overturned soil, presence of leaf litter, and occurrence of vegetation regrowth. Photographic standards were used to ensure age classifications were applied consistently.

Ground disturbance data were used to estimate the overall percentage of hunting blocks disturbed by pigs, and transitions between different age classes of disturbance. For the latter, transects were divided into 5 cm lengths and the predominant age class of disturbance for each segment contrasted between monitoring sessions. This allowed daily rates of new disturbance, re-disturbance (moving from older to newer disturbance classes) and recovery (moving from newer to older disturbance classes) to be calculated.

### Modelling ground disturbance by wild pigs

We estimated parameters for a model of ground disturbance by wild pigs originally described by Choquenot and Parkes [37]. The model can estimate the area of susceptible ground that is disturbed (*R*) based upon the rate at which undisturbed ground becomes disturbed (*m*) and the rate at which disturbed ground reverts to undisturbed ground (*n*).

$$R_{t}=R_{t-1}+m\;(1-R_{t-1})-n\;(R_{t-1})$$ (2)

To simulate variation in ground disturbance for a semi-arid rangelands ecosystem, Choquenot and Parkes [37] linked the rate of ground disturbance (*m*) to pasture biomass, assuming food availability would largely determine the propensity of pigs to feed on below ground resources. In this study because food availability was not measured directly, the effect of food availability on rates of ground disturbance (*m*) was evaluated using the relationship between ground disturbance and prevailing pig population density (*N*), expressed as a proportion of carrying capacity (*K*). Assuming that as above ground food resources are depleted at increasing levels of population density, the propensity for pigs to search for below ground food increases. Using the data from the ground disturbance transect monitoring, mean disturbance and recovery rates (*m* and *n* respectively, both estimated using the annual percentage of available ground that is either disturbed or recovers), were calculated for the three hunting blocks by using the data from transects that occurred within a hunting block. The rate of change was calculated between each of the four monitoring sessions, yielding nine estimates. The ratio of *N*/*K* between each monitoring session was estimated as the mean of the 1000 pig density model runs. The relationship between *m* and *n* and *N*/*K* was assessed with linear, exponential, power, log, and second-order polynomial models. The models were evaluated using *R*^2^ and *w* calculated from the model residuals.

### Linked pig-disturbance simulation models

To examine the potential cost-effectiveness of different culling strategies, we combined the pig population and ground disturbance models to simulate the effects of pig control on ground disturbance by hunting regimes that varied the frequency of hunting sessions from 0 to 3, 6, 9 or 12 months. Each hunting regime was modelled on a monthly interval for 30 years, with the results summarised as the mean and standard deviation of 1000 iterations of the model. Given the non-random distribution of ground disturbance at the study site (see results), the model predicted variation in ground disturbance for an unspecified “disturbance prone” proportion of the landscape, rather than for the overall study site.

For each hunting regime, pig density was modelled using Eq 1, with the initial and annual changes of *K* determined as a random selection from the log-normal distribution fitted to the initial population estimate, and with the pig population initialising at carrying capacity. As hunting efficiency was found to decline over the duration of the hunting programme (see results), variation in the density of pig removals (*C*) was included in models simulating pig control. In theory, a reduction in hunting efficiency over time reflects the effect declining pig density will have on the rate at which hunting teams locate and kill pigs. The relationship between estimated pig density and observed hunting efficiency was assessed with linear, exponential, power, log, and second-order polynomial models. The models were evaluated using *R*^2^ and *w* calculated from the models residuals. Hunting efficiency for any level of pig density within the model was generated by converting the estimate and standard error of *C* predicted by the chosen model to the geometric mean and standard deviation of a log-normal probability distribution from which a random value was generated.

In simulating ground disturbance, the initial level of ground disturbance was set at 15.7%, which was the average level measured across the four most disturbed transects during the initial monitoring session. The effect pig density has on ground disturbance was then simulated for each month using the model in Eq 2. The values of *m* and *n* were estimated from log-normal probability distributions for which the geometric mean and standard deviation parameters were estimated from the models fitted between *N*/*K* and *m* and *n*. Estimates of *m* and *n* were capped such that *m* and *n* had maximum values of 100%.

The accumulated cost of each hunting regime was also estimated. The average cost of hunting sessions undertaken over the course of the hunting programme was $23,800 New Zealand Dollars (NZD) (Auckland Council, unpublished data). This equates to an annual cost for a hunting regime based on 4 quarterly hunting sessions of $95,200, or $548.61/km^2^ (NZD).

## Results

### Hunting programme

**Fig 2:**
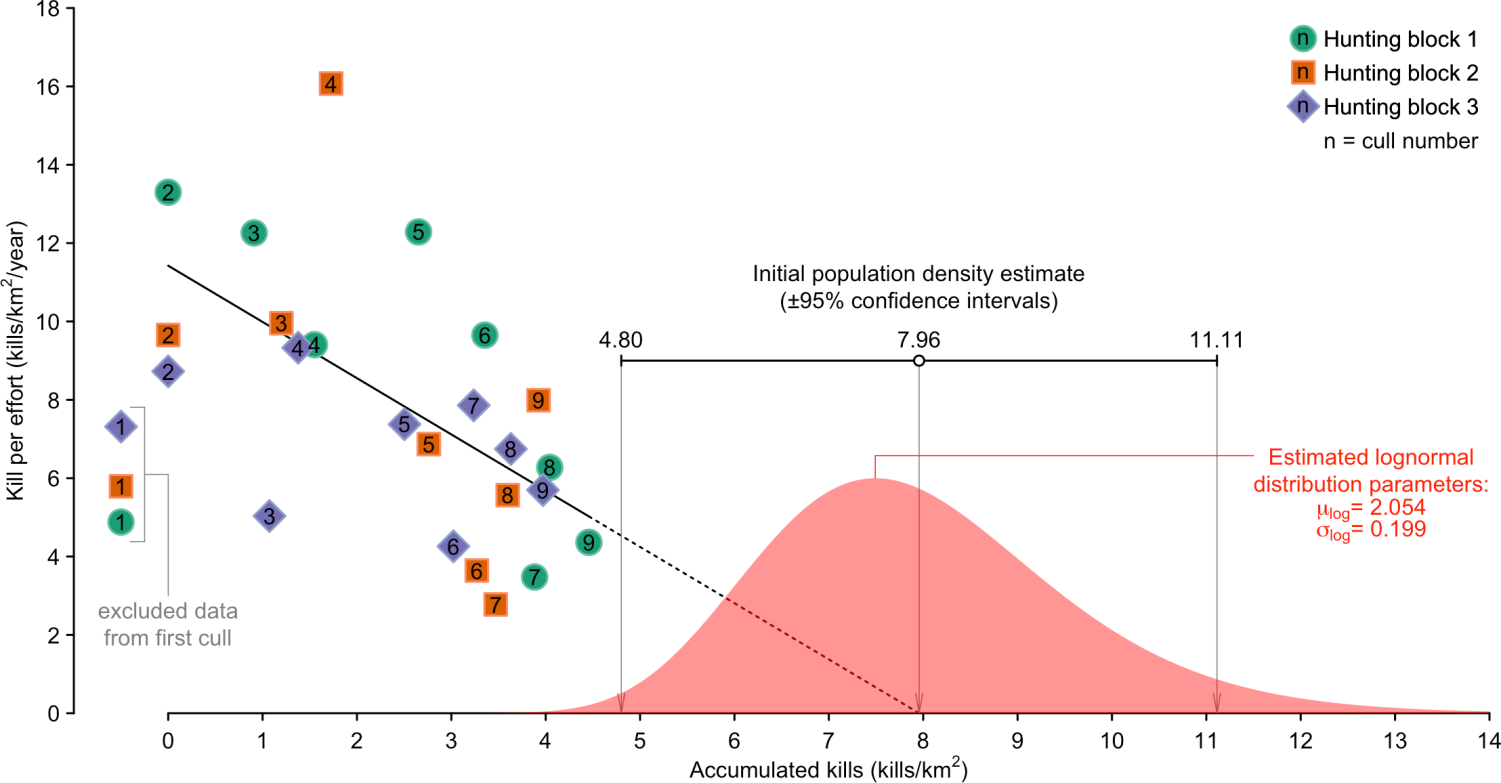
Estimate of the initial density of pigs within the Waitakere ranges based on a catch-effort methodology. The catch-effort population estimate and uncertainty was used to define a log-normal distribution to describe the likely range of possible initial population density. Estimates for the first hunting session have been excluded from the analyses, as they are likely to violate the assumption of constant catchability.

Hunting removed 895 pigs with cumulative kills/km^2^ of 5.56 for Block 1, 5.14 for Block 2 and 4.82 for Block 3. Preliminary examination of variation in hunting efficiency over the course of the hunting programme indicated no apparent difference between hunting blocks, with a general tendency for decline, but with the first hunting session for all blocks characterised by consistently low efficiency relative to this trend (Fig 2). Low hunting efficiency during the initial hunting session probably reflects the lack of familiarity hunting teams had with their assigned hunting blocks, and these estimates were excluded from subsequent analyses.

### Modelling pig density over the hunting programme

**Fig 3:**
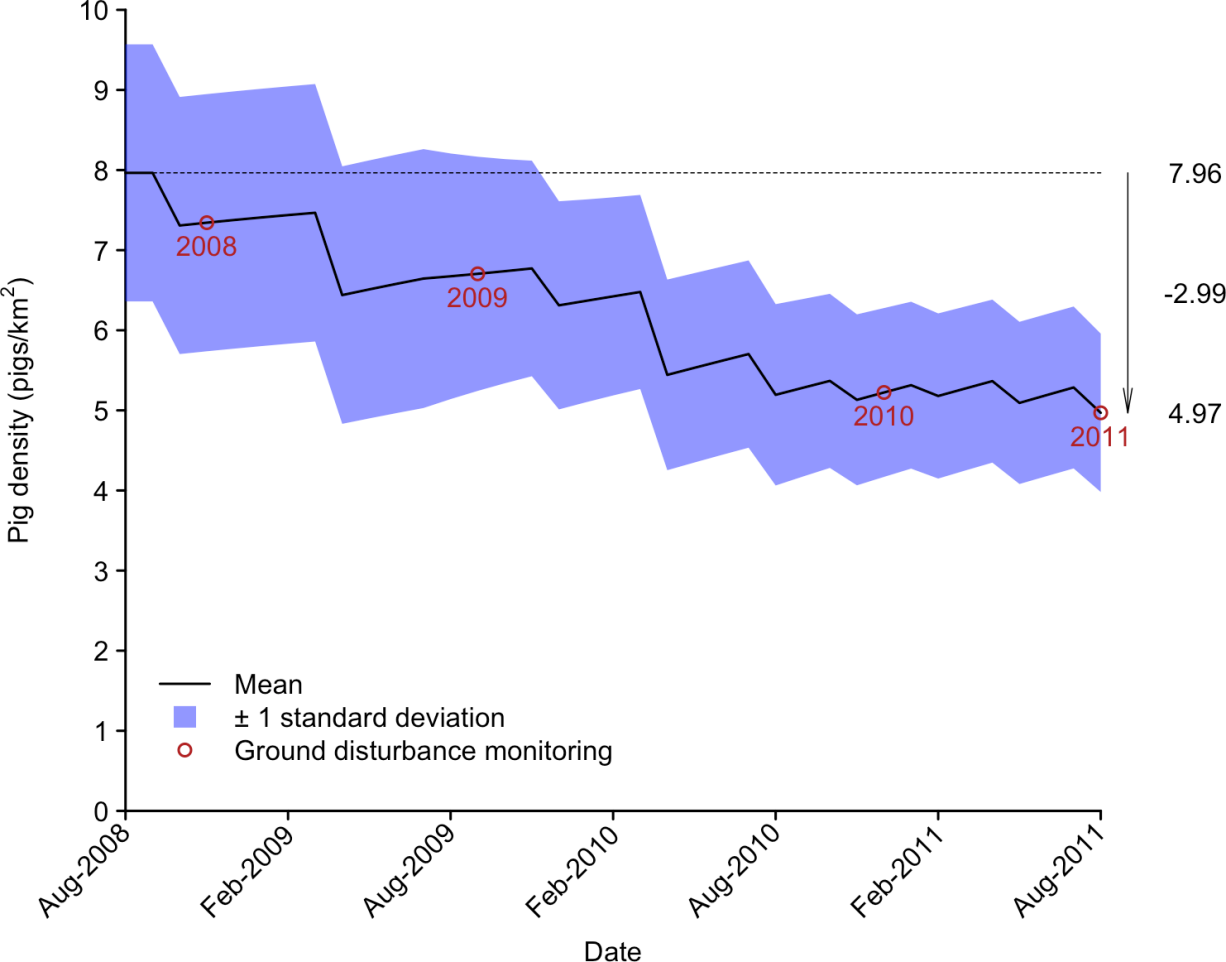
Projected changes in pig density over the duration of the hunting programme. The population projection is based on estimates of initial pig density, known removals and estimates of population growth from a simple logistic growth model. The mean and standard deviation are derived from 1000 model runs. The timing of ground disturbance monitoring sessions is also indicated.

The catch-effort method predicted an initial pig density of 7.96 pigs/km^2^, with 95% confidence intervals ranging from 4.80 pigs km^-2^ to 11.11 pig/km^2^ (Fig 2). The catch-effort assumption of linearity appeared to be held as the linear model provided by far the most parsimonious fit to the data (linear: *w* = 0.61, *R*^2^ = 0.36; second-order polynomial: *w* = 0.30, *R*^2^ = 0.38; exponential: *w* = 0.08, *R*^2^ = 0.31; log: *w* = 0.00, *R*^2^ = 0.13; power: *w* = 0.00, *R*^2^ = 0.08). Assuming the log-normal distribution of initial pig density is a reasonable estimate of the population density at *K*, the logistic growth population model projected a steady decline in pig density, with a final modelled density indicating a reduction of around 3 pigs/km^2^ (Fig 3).

### Ground disturbance

**Fig 4:**
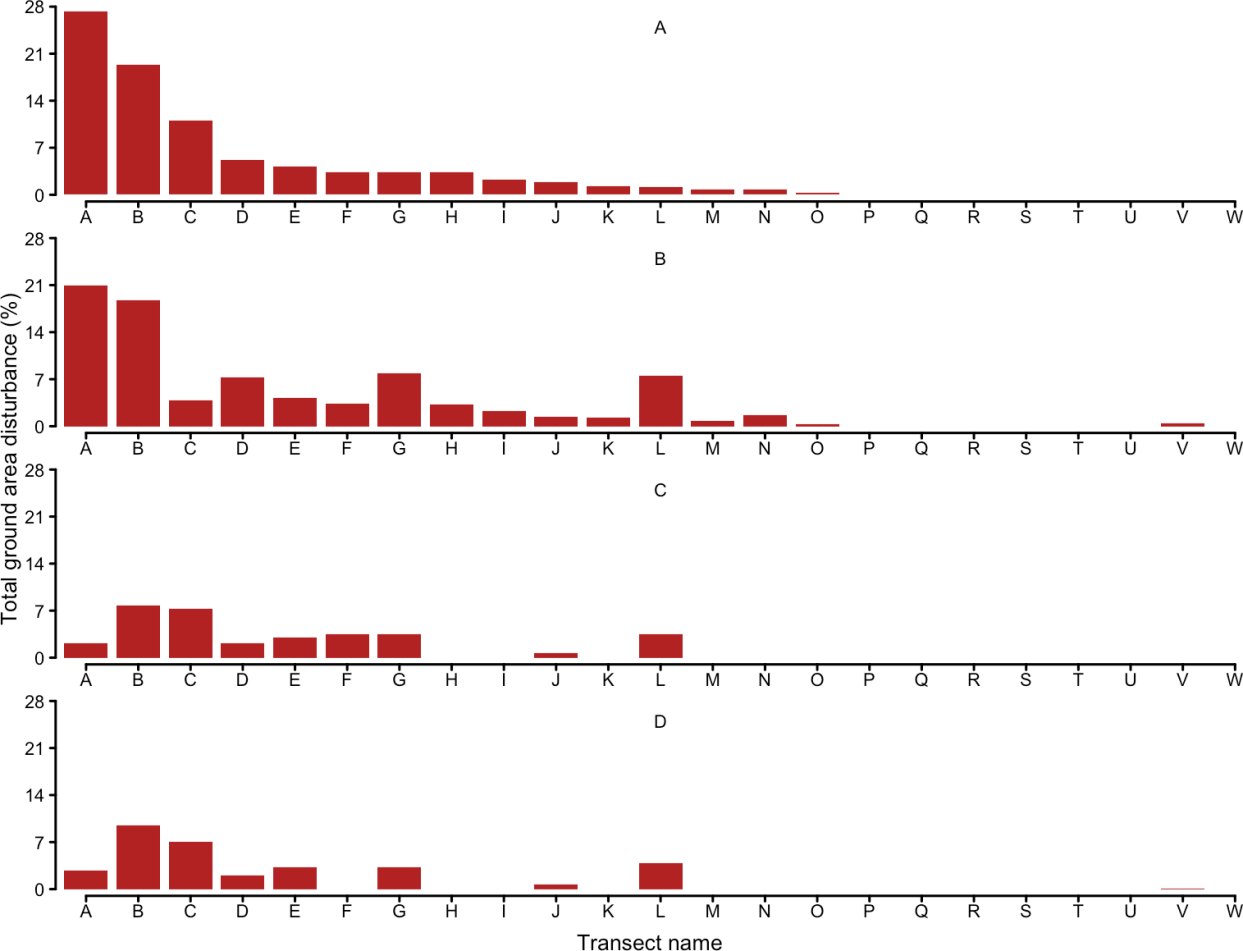
Changes in percentage ground area disturbed by wild pigs on 23 monitoring transects. Transect were (arbitrarily named A−W) at ground disturbance monitoring sessions in (A) 2008, (B) 2009, (C) 2010, and (D) 2011.

While the overall level of ground disturbance more than halved between the first and second half of the study (Fig 4), there was a strong tendency for the same transects to be disturbed regardless of the overall level of disturbance measured. For example, in the first monitoring session 74% of disturbance was recorded on four (17%) of the sampled transects (These transects were distributed across the three blocks). In subsequent monitoring sessions, the same transects sequentially represented 60%, 58%, and 66% of all disturbance measured.

### Modelling ground disturbance

**Fig 5:**
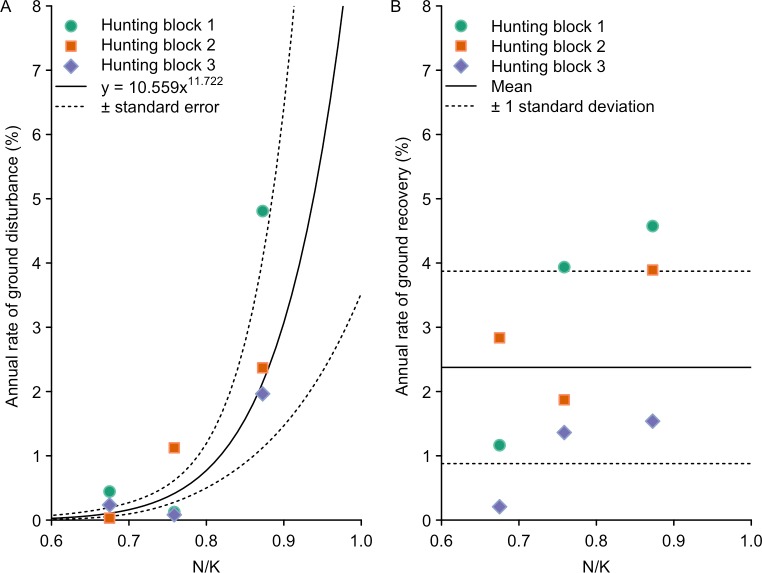
The relationship ground disturbance and recovery rates and pig density. (A) Annual rate of ground disturbance (*m*). (B) Annual rate of ground disturbance recovery (*n*). Pig density was a proportion of the estimated carrying capacity (*N*/*K*) for data combined across all three hunting blocks.

Preliminary examination of the data describing the relationship between the annual rate of ground disturbance (*m*) and relative prevailing pig density (*N*/*K*), suggested the rate of ground disturbance increased as prevailing pig density approached carrying capacity (Fig 5A). When fitting the linear and second-order polynomial models the intercept was set to zero to try and preclude models predicting negative ground disturbance rates with lower levels of *N*/*K*. Of the models predicting only positive rates of ground disturbance, the exponential and power models provided the most parsimonious fit to the data (exponential: *w* = 0.54, *R*^2^ = 0.63; power: *w* = 0.40, *R*^2^ = 0.60; linear: *w* = 0.07, *R*^2^ = 0.14). Although the exponential model had a slightly better fit to the data, we chose to use the very similar power model (Fig 5A). The power model had a slightly lower rate of increase, which we felt was more appropriate for extrapolating beyond our limited sample of nine data points. The power model also had the advantage of passing precisely through the origin, meaning that when pig density is zero the prediction of ground disturbance would also be zero.

Preliminary examination of the data describing the relationship between the annual rate of ground disturbance recovery (*n*) and prevailing relative pig density (*N*/*K*), indicated no systematic change in *n* (Fig 5B). Therefore, given the small sample size and the lack of an obvious trend we assumed that *n* was independent of *N*/*K*, and described the variation in *n* based on a mean rate of ground disturbance recovery of 2.4% per annum, with a standard deviation of 1.5% (Fig 5B).

### Linked pig-disturbance simulation models

**Fig 6:**
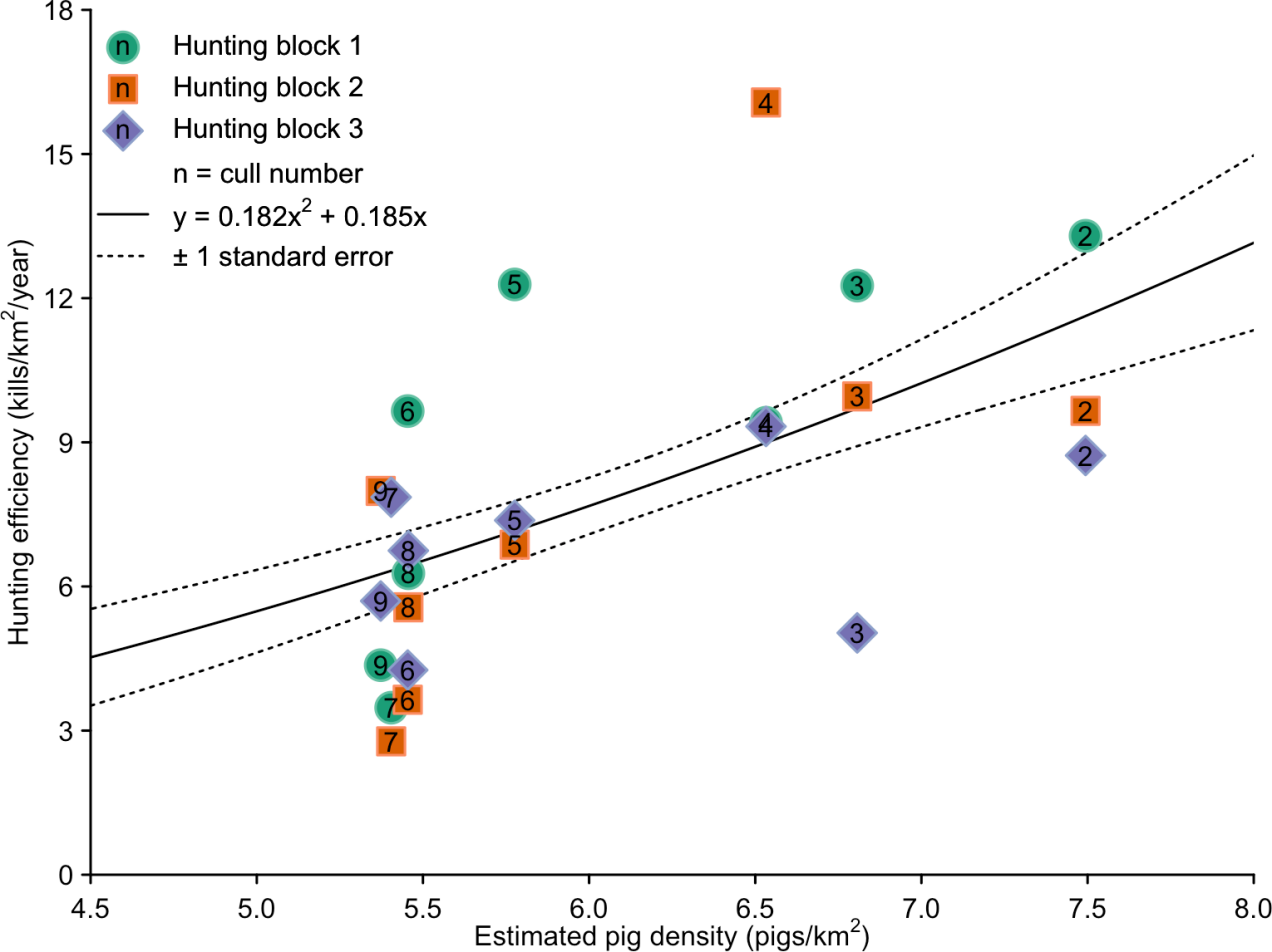
The relationship between hunting efficiency and density of pigs for each hunting session and hunting block.

When evaluating the relationship between pig density and hunting efficiency, the intercept of the linear and second-order polynomial models was set to zero to try and preclude models predicting negative efficiencies with lower pig densities. Of the models predicting only positive hunting efficiencies, the second-order polynomial model (Fig 6) provided the most parsimonious fit to the data (second-order polynomial: *w* = 0.33, *R*^2^ = 0.34; linear: *w* = 0.31, *R*^2^ = 0.26; power: *w* = 0.21, *R*^2^ = 0.31; exponential: *w* = 0.15, *R*^2^ = 0.29).

**Fig 7:**
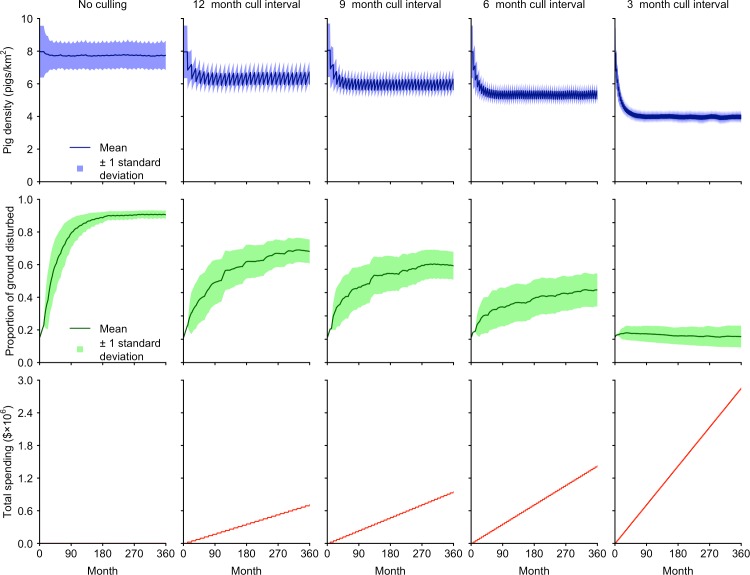
Predictions of pig density, ground disturbance, and accumulated cost for five different hunting regimes. The values for pig density and ground disturbance are the mean and standard deviation derived from 1000 model runs.

The stochastic simulation model comparing variation in pig density and ground disturbance over 30 years when hunting is imposed at various intervals shows more frequent pig control holds pig densities at lower average levels, and suppresses ground disturbance to a greater degree (Fig 7). The general trajectory of ground disturbance over the course of the simulations reflected an on-going decrease when hunting was implemented every 3 months, but stabilised when hunting was implemented every 6, 9 or 12 months. The residual density of pigs increased steadily as the frequency of hunting declined, with a concomitant increase in the mean rate of ground disturbance, and the proportion of disturbance-prone ground that was disturbed after 30 years. The predicted annual cost of each hunting regime increased proportionally with the frequency of hunting (Fig 7).

## Discussion

### Hunting efficiency

The decline in hunting efficiency over the course of the hunting programme (Fig 6) reflected the increasing time required to locate pigs to kill as density declined. This pattern is consistent with hunting teams operating as predators, and consequent kill rate taking the form of a functional response from predator-prey theory. According to this theory, the kill rate of a predator is determined by the time taken to locate prey (search time), then kill and consume prey (handling time) [38]. While handling time is usually considered constant for given prey types, search time increases as prey density declines [38]. The functional response has previously been used as a conceptual model for analysis and interpretation of variation in hunting efficiency for pigs being controlled by shooting from helicopters [39–40], and by ground teams [41–42].

### Pig population dynamics

The estimates of pig density at carrying capacity (Fig 2) was comparable to other studies in highly productive environments. Estimates of pig density vary widely, but appear to be linked to both the productivity of the habitat occupied, and the history of hunting or other control [43, 8]. The estimate of *K* derived in this study (7.96 pigs/km^2^), is comparable to the range of densities estimated for uncontrolled pig populations in temperate wetlands (8.0−17.5, [35]; 10.3, [44]), and subtropical floodplains and adjoining woodlands (6.1, [39]; 2.6−10.9, [45]; 1−>20, [46]). These environments support much higher densities of pigs than have been estimated for dry temperate forests or semi-arid rangelands, presumably because higher rainfall makes them significantly more productive [43].

The simple logistic model used to project changes in population density in this study assumed that migration was not a major influence on changes in pig abundance, rate of change being density-dependent with a maximum rate of *r*_*m*_ = 0.7. The estimate of *r*_*m*_ was based on studies of pig populations undertaken across a range of habitats in Australia that returned similar rates of increase for populations recovering from control operations. The maximum rate of increase for a wild animal population represents their genetically determined capacity to increase under conditions where no resources are limiting [47]. As such, habitat type is unlikely to influence *r*_*m*_, and the estimate used here for pigs is assumed to be a reasonable approximation. Similarly, the use of a simple logistic model implies that rate of increase reflects the instantaneous effect of variation in per capita food availability on population demography, with variation in food availability being relatively constant [47]. Dietary studies suggest that wild pigs in New Zealand forests gain the largest proportion of their food from above-ground vegetation [48]. In a climatically similar area of New Zealand (the Urewera Ranges), pigs have access to fallen fruit 6–9 months of the year, and year-round access to vegetation rich in carbohydrates. Fluctuations in pig density in these forests were modest and most likely related to short-term seasonal patterns of fruit fall [48]. Assuming the same types of food are utilised by pigs inhabiting the Waitakere Ranges, the logistic model provides a reasonable basis for projecting how pig populations would have responded to reductions in their density associated with the hunting programme.

### Ground disturbance

Reported levels of ground disturbance by wild pigs vary considerably. Highest reported disturbance percentages are for Florida (4.3–25.0%, [5]) and the Great Smoky Mountains National Park (15%, [13]). Other studies have found generally similar levels of percentage disturbance to that measured here, ranging from 1.1–13.3% [49–52]. In this study, the highest average percentage of ground disturbed was 6% (hunting block 1, monitoring session 1), but the overall level averaged around 4% across the entire study site. While this level of disturbance is generally consistent with the range of values reported in other studies, ground disturbance was strongly and consistently concentrated on a limited number of monitored transects, with little or no disturbance occurring elsewhere. The frequency of ground disturbance by pigs has been shown to be influenced by elevation [50, 52–53] proximity to drainage lines [54] and wetlands [51, 55], slope [56, 52], vegetation type [17], and rock cover [55].

Due to the lasting nature of disturbance in the landscape, observed levels of ground disturbance will be a function of previous and on-going rates of new disturbance and disturbance recovery rates. This means that the relationship between pig density and ground disturbance (the damage function) will reflect the effect that the short- to medium-term history of control has had on pig density, rather than a measure of current pig density *per se*. Future studies could focus on a more detailed scale of ageing ground disturbance patches to eliminate historical disturbance from their analysis. Hone [1] described a range of hypothetical damage functions linking equilibrium pest density to the area of damage they cause, depending on the relative rates of damage and recovery, the overall susceptibility of an area to damage, and the degree to which damage was proportional to pest density. In our study, long-term levels of ground disturbance increased exponentially with residual pig density, reflecting the rapid increase in the rate of ground disturbance as pigs approach carrying capacity. The acceleration in the rate of ground disturbance at pig densities approaching carrying capacity implies that pigs sought access to below ground food resources as density-dependent competition for above-ground resources intensified [37].

In contrast to the accelerating extent of ground disturbance with residual pig density shown in our study, Hone [8] described an asymptotic increase in the percentage of monitored plots with ground disturbance (positively correlated with the total area of ground disturbed) and the percentage of the same plots with pig dung (positively correlated with pig abundance), implying that the rate of new ground disturbance did not accelerate with increasing pig density as was the case in our study. This suggests little effect of density-dependent competition for above-ground food resources on the propensity of pigs to cause ground disturbance. However, Hone [8] indicated that the density of the pig population he studied was held well below carrying capacity by regular lethal control, suggesting pigs would rarely approach densities where food resources would become scarce.

### Controlling pigs to manage ground disturbance

The hunting regime achieved an initial reduction in ground disturbance that slowed over the final 18 months of the study. The level of disturbance within hunting blocks varied from around 1–6% over the first two monitoring sessions, declining to 1–2% over the final two monitoring sessions. This apparent reduction in the effectiveness of the hunting regime is probably due to the influence increasing population productivity had on the net effect of hunting. As pig density was reduced below carrying capacity (*K*), population productivity would be expected to increase because reduced density-dependent competition for food resources would elevate per capita reproduction and survival [57]. According to the logistic model of population growth used in the simulation modelling here, population productivity will continue to increase as pig density is progressively reduced from *K* to *K*/2, reducing the relative effectiveness of the hunting regime until densities below this level are achieved. The effect this has on the speed with which pig populations can be reduced is illustrated by the population density projections (Fig 3). While the frequency of hunting increased over the second half of the study, the projected decline in pig densities slowed noticeably. This decline would be expected to continue to slow until populations eventually stabilise at densities where all control effort is expended to simply remove the productivity of the population, with no further incremental reduction in underlying pig density [35, 40, 58].

The pig densities corresponding to 3, 6, 9 and 12 monthly hunting regimes are an example of these long-term stable outcomes (Fig 7). Of the four hunting regimes modelled, only a 3-month interval achieved a constant reduction in ground disturbance. For longer hunting intervals, pig populations stabilised at higher densities. This resulted in a stable level of ground disturbance, that while a reduction from the level expected when there was no hunting, would not result in the elimination of ground disturbance (Fig 7).

The annual cost of achieving progressively lower long-term pig densities, and commensurate decreases in ground disturbance indicate a strongly diminishing return on investment as lower levels of ground disturbance are targeted, supporting the diminishing returns principle [1]. This suggests that conservation managers concerned about the impact of ground disturbance on ecosystems need to consider carefully how much ground disturbance is too much, and prioritise their investment in pig control (relative to other conservation activities), accordingly [59]. The overall goal in controlling overabundant species (in our case pigs) is the reduction of their impact or damage rather than reducing their numbers *per se* [60–61]. However, to do so requires knowledge about the scale and consequences of the damage function [1]. If species density can be related to levels of damage (as demonstrated in our study), the relationship can be used to set thresholds for species control [59]. The model produced in this study provides a useful framework that links conservation of indigenous ecological communities to control inputs through the reduction of wildlife damage and evaluates the cost effectiveness of alternative control strategies in maintaining this goal.
